# Genomic Correlates of Immune-Cell Infiltrates in Colorectal Carcinoma

**DOI:** 10.1016/j.celrep.2016.10.009

**Published:** 2016-10-18

**Authors:** Marios Giannakis, Xinmeng Jasmine Mu, Sachet A. Shukla, Zhi Rong Qian, Ofir Cohen, Reiko Nishihara, Samira Bahl, Yin Cao, Ali Amin-Mansour, Mai Yamauchi, Yasutaka Sukawa, Chip Stewart, Mara Rosenberg, Kosuke Mima, Kentaro Inamura, Katsuhiko Nosho, Jonathan A. Nowak, Michael S. Lawrence, Edward L. Giovannucci, Andrew T. Chan, Kimmie Ng, Jeffrey A. Meyerhardt, Eliezer M. Van Allen, Gad Getz, Stacey B. Gabriel, Eric S. Lander, Catherine J. Wu, Charles S. Fuchs, Shuji Ogino, Levi A. Garraway

(Cell Reports *15*, 857–865; April 26, 2016)

Table S3 has been updated. The updated version includes all somatic mutations used in our analyses.

